# The contemporary design of endovascular aneurysm stent-graft materials: PTFE versus polyester

**DOI:** 10.3389/fsurg.2022.984727

**Published:** 2022-08-16

**Authors:** Niamh Hynes, Yogesh Acharya, Sherif Sultan

**Affiliations:** ^1^ CURAM SFI Centre for Medical Devices, Biomedical Sciences, National University of Ireland Galway, Galway, Ireland; ^2^ Department of Vascular & Endovascular Surgery, Western Vascular Institute, Galway University Hospital, Galway, Ireland; ^3^ Department of Vascular & Endovascular Surgery, Galway Clinic, Galway, Ireland

**Keywords:** endograft complications, aorta—remodeling, polyester, EVAR, aortic compliance/distensibility, polytetrafluoroethylene

## Abstract

Endovascular aneurysm repair of the abdominal aorta (EVAR) and of the thoracic aorta (TEVAR) have revolutionised therapeutic strategies in the management of aortic pathology, and endovascular repair is now an established and attractive alternative to open surgical repair (OSR) due to its superior short-term safety profile. However, opinions are divided regarding its long-term cost-effectiveness, which is reflected in the controversial NICE guidelines on abdominal aortic aneurysm (AAA) repair published in 2018, which advised against EVAR for elective aortic repair due to high secondary intervention rates and resultant associated costs. There is no doubt that OSR continues to have a valuable role to play in aortic repair, but it is not universally applicable, especially in older and sicker patients. Therefore, we should not dismiss EVAR and TEVAR without examining the reasons for long-term failure, and the most obvious starting point is stent graft material properties. Polytetrafluoroethylene (PTFE) and polyester are the two most common stent-graft materials; however, there has been no objective comparison of PTFE and polyester stent-graft post-procedural outcomes in EVAR and TEVAR, or even OSR. This lack of definitive data on different stent-graft materials and their configuration necessitates a comprehensive review to elucidate the post-procedural outcome in terms of endograft failure, cardiovascular events, and aortic-related mortality and morbidity.

## 1. Introduction

Aortic disease management, either with surgical grafts or endovascular devices, has undergone minimal development since inception over 70 years and 35 years ago, respectively. Both open and endovascular grafts are “passive”, one-type-fits-all devices that do not consider anatomical location or the underlying pathology (aneurysm, dissection, trauma) and do not reinstate the aorta's regeneration, biomechanical or physiological functions, resulting in long-term major adverse cardiovascular (CV) events and high reintervention rates.

Open aortic repair (OAR), first introduced in the 1950s, has historically been the gold standard technique for treating aortic aneurysms and dissections. In OAR, a prosthetic [Polyester or Poly Tetra Fluoro Ethylene (PTFE)] surgical graft is used to **replace** the affected aortic segment. Depending on which body cavity (abdomen, thorax or mediastinum) or how many body cavities are opened, OAR can be a challenging, highly-invasive procedure unsuitable for older and those with extensive co-morbidities. The risks associated wth OAR prompted the development of endovascular procedures in the late 90s. Endovascular became the preferred choice given its lower invasiveness, shorter hospital stays and quicker recovery times. In endovascular procedures, a metal scaffold covered by fabric (endograft) is inserted inside the aorta to **exclude** the diseased wall from the circulation. However, although endovascular repair became the treatment of choice in most cases, it is not feasible in all cases due to the variety of adverse anatomical features (e.g., sufficient length of the normal aorta for an implant landing zone, vessel tortuosity, access vessel calcification etc.). Despite such exceptions, endovascular therapy was quickly disseminated into clinical practice. However, following the adoption of endovascular techniques, questions about its durability started to arise, and guidelines issued by the UK's National Institute for Health and Care Excellence (NICE) in 2020 recommend against endovascular aortic repair (EVAR) as first-line management of elective infrarenal aortic aneurysm, based on surveillance costs and high re-intervention rates (1). The NICE guidelines sent significant ripples through the clinical community. Still, they have provided an opportunity for reflection and impetus to consider if certain aspects of EVAR could be improved, such as stent-graft materials.

Polytetrafluoroethylene (PTFE) and polyester are the two most common stent-graft materials; however, there is no objective evidence comparing their relative effectiveness in abdominal aortic aneurysm (AAA) repair. In this review, we consider the influence of the contemporary stent-graft materials and their configurations on short and long-term post-procedural outcomes amongst patients undergoing EVAR for Abdominal Aortic Aneurysm (AAA). The primary aim is to compare the available contemporary stent-graft materials, PTFE versus polyester, on aneurysm-related mortality and cardiovascular outcomes. Secondary aims include graft-specific complications and reintervention rates. Subgroup analysis was planned to consider variable graft configurations on clinical outcomes.

## 2. Methods

This project was undertaken per the PRISMA guidelines (2) and recommendations described in the Cochrane Handbook for Systematic Reviews of Interventions (3).

### 2.1. Criteria for considering studies for this review

#### 2.1.1. Types of studies

We considered randomised controlled trials (RCTs) and controlled clinical trials (CCTs) comparing patients undergoing EVAR treated with endografts made from polyester to patients treated with endografts made from PTFE for AAA for inclusion in the review. We placed no limitations on publication date, language, or status.

#### 2.1.2. Types of participants

All participants with AAAs undergoing EVAR diagnosed using conventional methods, such as computed tomography (CT) or magnetic resonance imaging (MRI), or both, were to be included in the review. We planned to consider people with a primary AAA of any morphology (e.g. fusiform, in which the entire circumference of the aneurysmal portion of the aortic wall is dilated, as opposed to a saccular aneurysm in which there is an eccentric outpouching of the aortic wall). We did not consider aneurysm formation post-aortic dissection.

#### 2.1.3. Types of interventions

We planned to include all studies comparing endovascular repair with a polyester-based endograft versus a PTFE-based endograft. For endovascular repair, several devices are available, and we planned to include all device types.

#### 2.1.4. Types of outcome measures

The selection of primary and secondary outcomes was guided by the Society for Vascular Surgery (SVS) reporting standards for thoracic endovascular aortic repair (4). We planned to report outcomes on time points such as 30 days, 12 months and five years unless otherwise stated.

##### 2.1.4.1. Primary outcomes

(1) aneurysm-related death (including rupture and death within 30-days of procedure) and (2) major adverse cardiovascular events (myocardial infarction, heart failure, arrhythmia, cardiovascular death).

##### 2.1.4.2. Secondary outcomes

(1) endoleak, (2) aneurysm sac expansion, (3) reintervention, (4) graft infection, (5) thrombosis, (6) post-implantation syndrome, and (7) all-cause mortality.

### 2.2. Electronic searches

Systematic searches of the following databases for RCTs and CCTs were undertaken without language, publication year or publication status restrictions.

• Cochrane Vascular Specialised Register *via* the Cochrane Register of Studies (CRS-Web) (searched April 26, 2021)
• Cochrane Central Register of Controlled Trials (CENTRAL; 2021, issue 3) *via* the Cochrane Register of Studies Online (CRSO)
• MEDLINE (Ovid MEDLINE Epub Ahead of Print, In-Process & Other Non-Indexed Citations, Ovid MEDLINE Daily and Ovid MEDLINE) (searched April 26, 2021)
• Embase Ovid (searched April 26, 2021)
• CINAHL Ebsco (searched April 26, 2021)
• AMED (searched April 26, 2021)

The following trial registries were also searched on April 26, 2021.

• World Health Organization International Clinical Trials Registry Platform (who.int/trialsearch)
• ClinicalTrials.gov (clinicaltrials.gov)

### 2.3. Searching other resources

References of relevant articles retrieved from the electronic search for additional citations were also searched.

### 2.4. Selection of studies

Two review authors (NH and SS) independently screened all titles and abstracts identified from the literature searches to identify those that met the inclusion criteria. We retrieved the full text of studies identified as potentially relevant by at least one author. The same review authors independently screened the full-text articles for inclusion or exclusion. We resolved any disagreements by discussion or, when necessary, we consulted a third review author (YA). The screening and selection processes are presented using the adapted PRISMA flowchart.

## 3. Results

**Figure 1:**
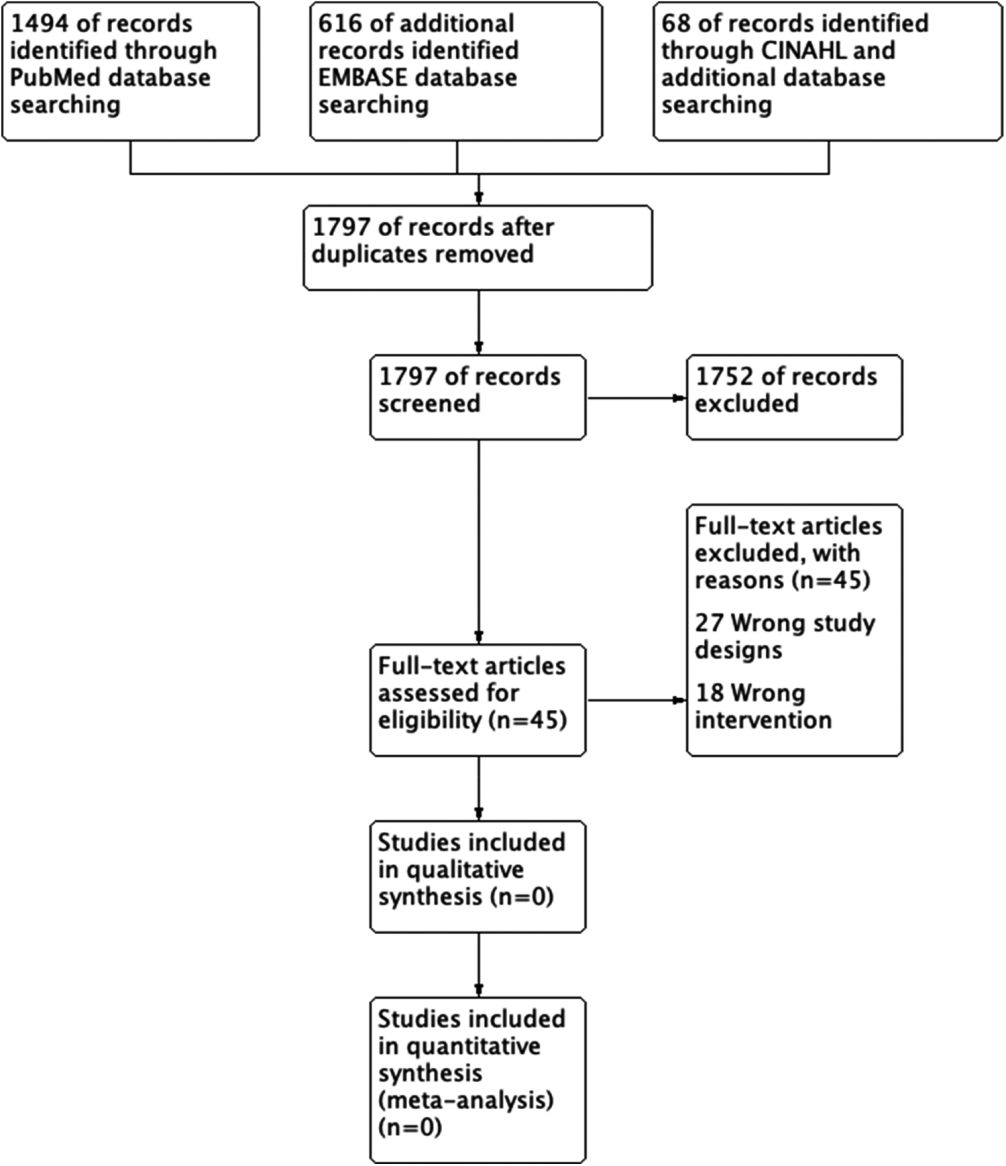
PRISMA study flow diagram.

The search generated 2,178 references. A total of 381 duplicates were identified and removed. The titles and abstracts of the remaining 1,797 studies were then reviewed. Of the 1,797 studies reviewed, we only carried 45 studies to full-text review and included or excluded studies based on study type and PICO (Figure 1). However, none of the studies met the inclusion criteria, and we did not find any RCT or CCT that compared stent-graft materials in endovascular AAA repair.

## 4. Discussion

We did not find any RCT or CCT that considered comparing PTFE and Polyester materials and their influence on post-procedural outcomes following EVAR. However, we did find ample evidence that endovascular repair has adverse effects, especially cardiac and aortic dysfunction. It is likely that this is a function of the overall endograft contrast rather than simply related to whether the material used is PTFE or polyester. Considering the impact of aortic disease and the expense of aortic repair, suboptimal outcomes warrant further reflection.

Aortic diseases, including aneurysms and dissections, are a leading and growing cause of death worldwide: Globally, the number of aortic aneurysm deaths increased to 172,426 in 2019, a rise of 82.1% compared with 1990 (5). The climbing death rate is even more pronounced in developing countries, with an increase in median death rate (per 100,000) of 0.71, three times higher than in the developed world, where it is 0.22 (6). Mortality is especially evident in those who present with Acute Aortic Syndromes (AAS). Despite an almost universal fall in CV deaths over the last two decades, the incidence of AAS mortality has not fallen in the past 40 years: those with AAS have more than double the mortality rate of age-matched controls at 5, 10, and 20 years post AAS (7). They have a two-to-threefold increased risk of non-aortic CV death, any first-time non-fatal CV event, and first-time heart failure (8). Even those that survive the initial acute aortic event continue to have a substantial risk of aortic death, aortic event, aortic intervention, and first-time diagnosis of aortic aneurysm (9).

The global aortic aneurysm market was valued at €2.3 billion in 2018 and is expected to register a compound annual growth rate (CAGR) of 8.6% from 2019 to 2026 (10). This translates into an increase in aortic repairs from 219,664 in 2021 to a projected >400,000 repairs in 2030. Despite perceived progress in diagnostic and therapeutic techniques, the economic burden of aortic disease is growing (11–14). In 2020, McClure et al. analysed the healthcare resource use for thoracic aortic dissections and aneurysms in Canada, which reached €430 million over a 13-year period (15). Cost expenditures to treat thoracic aortic disease escalated in an upward projection, with yearly total hospital costs significantly increasing beyond the rate of inflation over the period. The use and cost of posthospital healthcare resources were also considerable. Home care services alone were used by 40% of patients, and the one-year hospital readmission rate was 22%. Extrapolating these numbers to the European population, we could reach a tremendous yearly burden of >€1.7 billion for thoracic aortic diseases only. The abdominal aortic device market is 3.5 times bigger than the thoracic device market. (There were 169,261 Abdominal procedures versus Thoracic 46,476 procedures in 2021.) A notable percentage of these costs can be attributed to the limitations of currently used devices

### 4.1. Current graft materials

Synthetic grafts for OAR fail to replicate the elasto-mechanical characteristics of the native arterial tissue. The consequent lack of adequate compliance leads to a cascade of hemodynamic and biological alterations adversely affecting cardiovascular homeostasis, especially when implanted near the heart (16). Proximal prosthetic graft replacement of the ascending aorta amplifies circumferential strain in the descending thoracic aorta, modifies energy propagation to the distal aorta and contributes to distal aortic disease manifestation (17).

These effects are even more pronounced with endovascular devices. Since the first commercially available devices were launched, aortic endografts have undergone only modest enhancements in stent material, graft fabric, fixation method, deployment mechanism, and flexibility. However, the underlying principle has not changed substantially. Both the metallic skeleton and the graft materials reduce aortic compliance, causing a mismatch in the physio-mechanical properties between the native and stented aorta (18). The materials in current endografts are designed to enhance the durability of the graft and reduce the risk of endoleaks, but endografts have biomechanical properties that are several orders of magnitude stiffer than the native aorta (19). Aortic compliance is critical to reducing the impedance and workload of cardiac ejection. Acute stiffening of the aorta following endovascular procedures results in acute elevated pulse pressure, hypertension, decreased coronary artery perfusion, and heart failure (20). The challenges of poor compliance, endovascular aortic device failure and the need for reinterventions undermine the cost-effectiveness of endovascular repair. The endovascular approach was driven by a clinical need to treat patients unfit for open repair. Thus, the original clinical needs remain unmet.

The aortic structure and function vary considerably along its length, yet devices (open and endovascular) do not vary by anatomical location or address the variability in physiological requirements. The differences in functionality of the aorta correspond with embryological origin (21, 22). The proximal aorta arises from the cardiac neural crest as part of the left ventricular outlet tract. It has a combined need for capacitance (enhanced elasticity) and the need to propel blood forward. The distal aorta, beyond the level of the left subclavian artery, arises from the mesoderm and is associated with more muscular contraction. These differences in compliance and function alter device function relative to location. Using patient-specific FSI models (19) which used 4D MRI Dual VENC sequences (23) to quantify patient- and location-specific aortic compliances, we demonstrated that the degree of oversizing needed to prevent endoleaks in the proximal aorta was more than double that required in the distal aorta. We also found that other factors, such as ageing of the aorta, whereby the collagen transition strain and elastin content are decreased, influence the percentage oversizing. This is significant because as the percentage oversizing increases, the device compliance dramatically decreases, but clinicians or device manufacturers are not considering these factors.

In recent years, some devices have been developed, providing important learnings to the community. The Personalised external aortic root support (PEARS) device by Exostent prevents aortic enlargement and rupture by being placed around the ascending aorta and is manufactured using advanced medical imaging and computer-assisted 3D printing. Studies have shown that this biomechanical support appears to modulate tissue function and promotes recovery of the microstructure of the media. However, the PEARS device requires open surgery, and its application is limited to a small number of patients with Marfan syndrome and related genetic conditions with early dilatation (40–45 mm rather than >55 mm). The Multilayer flow modulator (MFM) device by Cardiatis is an Endovascular 3D mesh with compliance more similar to that of the native aorta. The MFM was the first device that focused on manipulating flow rather than looking at anatomy. In this way, compliance, endothelialisation, and reduction of thrombus formation are also targeted, all of which reduce peak wall stresses while simultaneously enhancing wall strength and promoting healing. However, the MFM was inappropriately disseminated and has suffered in terms of reputation. We found that this device performs well for aortic dissections but not for large chronic aneurysms, where it cannot achieve modulation beyond a certain aortic size (9, 24, 25).

Despite clear limitations, both the PEARS and MFM devices have taught us that biomechanical support and optimal compliance can positively affect tissue modulation. Biomechanical support changes gene expression of the aortic tissue to promote aortic wall modulation, which in turn improves biomechanical function. This positive feedback loop leads to tissue healing. With these passive devices, modulation was possible when the disease was in its early stages; however, since aortic disease is predominantly asymptomatic and, most patients present when their disease is beyond the early stages, more active approaches are required to achieve repair and restore tissue function.

### 4.2. PTFE versus polyester

There have been limited non-randomised studies which have compared PTFE and Polyester in aortic endografts. Using pulse wave velocity (PWV) as a surrogate marker to demonstrate changes in stiffness following EVAR, Kadoglou et al. (26) showed that post-EVAR with polyester endografts, there could be a threefold increase in PWV compared to PTFE. Differences have also been reported between these materials in open aortic surgery. PTFE endografts had been reported to offer significantly stronger resistance to dilatation than polyester-based endografts, albeit this advantage is lost over time (27).

There are some other effects that materials may have beyond biomechanical, such as the development of the post-implantation syndrome (PIS). PIS has been reported in up to two-thirds of the patients following EVAR (28). and can result in acute liver and/or multiple-organ failure (29–34). However, the high rate of PIS reported is likely a consequence of a robust diagnostic criteria. The symptoms and signs (high fever, leukocytosis, and elevated serum CRP and interleukin (IL)-6) are often seen as a systemic post-operative response and it can be difficult to distinguish these from PIS and some authors have used other surrogate markers to demonstrate the pathological consequences of PIS. Ito et al. (28), Voûte et al. (10), and Sartipy et al. (11) implicated polyester-based endografts in developing postoperative pyrexia, PIS, and extended hospital stay post-EVAR compared to the PTFE-based endografts. Endografts with woven polyester are thought to be associated with a more robust inflammatory response which results in endothelial damage. Ferreira et al. (12) suggested a possible link between PIS and increased cardiovascular mortality. Also, polyester implanted grafts result in an augmented inflammatory response, mainly due to IL-8 serum levels. IL-8 is a neutrophil chemoattractant that exerts different pro-tumoural functions and plays a vital role in tumour progression and metastasis. This may elucidate the probable malignant potential of polyester-based endografts (13, 14). However, there are currently no controlled studies to substantiate these findings.

### 4.3. Limitations

A significant limitation of our review is that we only sought to include RCTs and excluded other clinical studies. Observation studies and non-randomised trials could have been an essential source of information given the non-availability of RCTs and/or CCTs. As we could not find any RCTs based on our study objectives, we could not reach a consensus regarding the implications of the specific stent-graft materials in the post-procedural outcomes following EVAR. Of consideration is that stent-graft material alone is challenging to investigate because of confounding factors. The evidence regarding the possible post-procedural outcomes implicated in the stent-graft materials could also be attributed to the device design, shape, and the presence of an exoskeleton versus an endoskeleton.

Overall, it has been demonstrated that the currently available endografts, regardless of the stent-graft materials, are less compliant than the native aorta, and they fail to simulate the elasto-mechanical qualities of the native aorta due to insufficient compliance. Given the increasing evidence of adverse hemodynamic alteration post-EVAR, the best solution in the short term could be to reduce the stented length of the aorta. At the same time, in the longer term, encourage continuous improvement in stent-graft materials and design.

We undertook this systematic review to investigate if device materials related to adverse outcomes, i.e., PTFE vs polyester; however, not surprisingly, we did not find any RCTs or CCTs which were structured or powered to answer these specific questions. Regardless, we found experimental and observational studies that support the hypothesis that graft materials and lack of compliance adversely affect cardiac function. The paucity of RCTs is a motivation for undertaking an RCT, which owing to the variation in specialisation across aortic centres, may require us to utilise cluster-randomisation. However, the logistical difficulties of undertaking an RCT should not be underestimated. International collaboration is necessary to recruit significant enough numbers and obtain sufficient funding. Before this, the next logical step is to undertake a patient-level meta-analysis to inform specific trial outcomes. Other means of collecting evidence, such as a prospective registry—based randomised controlled trial (RRCT) is possible. RRCTs are growing in popularity, especially in Scandinavian countries where large national registries already exist, and have been applied to the assessment of cardiovascular therapies (15). Using existing data reduces cost and administrative burden, and as RRCTs do not have inclusion and exclusion criteria like RCTs, the outcomes can more readily be applied to real-world scenarios, which make them especially attractive for aortic diseases.

## Data Availability

The raw data supporting the conclusions of this article will be made available by the authors, without undue reservation.
